# Volatile Compounds in Honey: A Review on Their Involvement in Aroma, Botanical Origin Determination and Potential Biomedical Activities

**DOI:** 10.3390/ijms12129514

**Published:** 2011-12-20

**Authors:** Christy E. Manyi-Loh, Roland N. Ndip, Anna M. Clarke

**Affiliations:** 1 Microbial Pathogenicity and Molecular Epidemiology Research Group, Department of Biochemistry and Microbiology, Faculty of Science and Agriculture, University of Fort Hare, P/Bag X1314, Alice 5700, South Africa; E-Mails: 200902845@ufh.ac.za (C.E.M.-L.); rndip@ufh.ac.za (R.N.N.); 2 Laboratory for Emerging Infectious Diseases, Department of Biochemistry and Microbiology, Faculty of Science, University of Buea, Box 63, Buea, Cameroon

**Keywords:** honey, natural product, VOCs, aroma, floral origin, biomedical activities

## Abstract

Volatile organic compounds (VOCs) in honey are obtained from diverse biosynthetic pathways and extracted by using various methods associated with varying degrees of selectivity and effectiveness. These compounds are grouped into chemical categories such as aldehyde, ketone, acid, alcohol, hydrocarbon, norisoprenoids, terpenes and benzene compounds and their derivatives, furan and pyran derivatives. They represent a fingerprint of a specific honey and therefore could be used to differentiate between monofloral honeys from different floral sources, thus providing valuable information concerning the honey’s botanical and geographical origin. However, only plant derived compounds and their metabolites (terpenes, norisoprenoids and benzene compounds and their derivatives) must be employed to discriminate among floral origins of honey. Notwithstanding, many authors have reported different floral markers for honey of the same floral origin, consequently sensory analysis, in conjunction with analysis of VOCs could help to clear this ambiguity. Furthermore, VOCs influence honey’s aroma described as sweet, citrus, floral, almond, rancid, *etc*. Clearly, the contribution of a volatile compound to honey aroma is determined by its odor activity value. Elucidation of the aroma compounds along with floral origins of a particular honey can help to standardize its quality and avoid fraudulent labeling of the product. Although only present in low concentrations, VOCS could contribute to biomedical activities of honey, especially the antioxidant effect due to their natural radical scavenging potential.

## 1. Introduction

Honey is a nutritious food, with economical importance worldwide, and is the most important primary product of beekeeping (utilizing the honeybees *Apis mellifera* L.) used by humankind since ancient times [1]. It is mainly a supersaturated sugar solution, with more than 95% of its dry mass consisting of sugar and water, although different valuable nutrients such as vitamins, minerals, enzymes, flavoring organic compounds, free amino acids and numerous volatile compounds constitute minor components [2]. However, it is this smaller fraction of the overall composition that is responsible for honey’s organoleptic and nutritional properties.

Nevertheless, the composition of honey is tightly associated to its botanical source and also to the geographical area from where it originated, because soil and weather determines melliferous flora [3]. With regard to their origin, honey could be classified as floral when it is derived from the nectar of flowering plant or non-floral (Honeydew) when it is derived from sweet deposits secreted by living parts of plants or excreted onto them by sap-sucking insects [4,5]. According to Polish and European present law, a third category known as mixed honey (Honeydew and nectar) is equally available [6]. Consequently, there is disparity in the chemical composition between floral and honeydew honey [7]. Physicochemical characteristics such as pH, acidity, proline content, ash content, color, and electrical conductivity have been considered to be useful characteristics for differentiating floral honeys from honeydew honeys [8].

More elaborately, honeydew honeys are darker and also have a higher pH and greater acidity which could be attributed to the characteristic high acetic acid concentration, even though the formation of this compound by microbial metabolism cannot be ruled out [9]. Furthermore, variations exist in the volatile compound composition as well as the anti-oxidant potential. As suggested, the presence of certain compounds such as 1-(2-furanyl)-ethanone, 2, 3-butanediol and 3-hydroxy-2-butanone and 1-hydroxy-2-propanone could be used to discriminate between these honey types [7]. Unifloral honeys differ from each other, among other features, in volatile organic composition which influences remarkably the individual sensory characteristics of each honey type [10].

More than 600 compounds have been identified as honey volatiles in different chemical families, originated from various biosynthetic pathways [11]. Generally, volatile organic compounds (VOCs) could be derived from the plant or nectar source, from the transformation of plant compounds by the metabolism of a bee, from heating or handling during honey processing and storage, or from microbial or environmental contamination [12–14]. The chemical families into which the volatile compounds in honey belong include: hydrocarbon; aldehyde; alcohol; ketone; acid; ester; benzene and its derivatives, furan and pyran; norisoprenoids; terpenes and its derivatives and sulphur; and cyclic compounds [15].

Nonetheless, the composition of VOCs in honey is influenced by both nectar composition and floral origin, which could also be associated with the honey’s geographical origin [16]. In addition, variations occur in the level of volatile components found in honey during storage as a result of the temperature at which it is exposed and also the period of exposure. These changes in heated or stored honey have been attributed to two principal causes: compounds that are heat labile and may be destroyed, and volatile compounds produced by non-enzymatic browning (Maillard reaction). Moreover, in a study conducted by Castro-Várquez *et al*. [17], there was a decrease in the concentrations of terpene derivatives and methyl anthranilate in contrast to an increase in concentration of linalool, linalool oxides and dien-diols in stored citrus honeys.

In this paper, we explore the analysis of the volatile fraction of honey as a method for assessing the aroma and floral origins as well as their antimicrobial potentials.

## 2. Isolation Techniques for Volatiles in Honeys

The specificity of honey products, *i.e.*, flavor, aroma, color and texture, depends predominantly on the type of flowers, or plants, from where bees take the nectar, or the honeydew, to produce honey. Some other factors have a lesser influence on the properties of honey, *i.e.*, the physiology of bees and their foraging habits, the climatic conditions (temperature, humidity) and the post-collection processing. Naturally, bees will forage any type of flower they can reach, and in consequence the honey produced mostly has a blend of flavors and is commonly sold in the market simply as honey or mixed-flower honey [18]. In this light, honey is a highly complex substrate to analyze, since it contains many volatile components with different chemical structures of low concentration in a sugar matrix where polar substances are the major components [19].

Due to its low concentration, it is necessary to remove the sugars which are the major components in honey before isolating the volatiles [20]. The extraction of these compounds prior to analysis by gas chromatography-mass spectrometry (GC-MS) has been carried out by numerous techniques, all of which have various advantages and disadvantages [21]. However, the composition of the honey volatiles show strong variability according to the extraction systems, since the volatility and polarity of each compound significantly affects the recovery percentage [22].

Some of these methods for instance, hydrodistillation (HD), liquid–liquid extraction (LLE), simultaneous steam distillation extraction (SDE) or Likens-Nickerson simultaneous distillation extraction (LNSDE) and micro-simultaneous steam distillation-solvent extraction (MSDE) utilize heat; however, heat treatment can lead to the formation of furan and pyran derivatives due to the effect of heat on sugar or amino acids (non-enzymatic browning reaction/Maillard reaction) [23]. Moreover, sensitive compounds are easily oxidized or decomposed and new components arise that do not belong to the aroma of honey [21].

Furthermore, the aforementioned methods and ultrasonic solvent extraction (USE) equally implement the use of solvents which is disadvantageous, even though the most commonly used low polarity solvents extract neither water nor sugar from honey [24]. USE aids extraction by significantly reducing extraction times and greatly improving extraction efficiency since the mechanical effect of the ultrasound would agitate the extraction solvent providing a greater penetration of the solvent into the matrix via cavitation [25]. The use of chemical solvents can solubilise non-volatile compounds and contaminate the GC port. Also, some analytes can be masked by the solvent and elude the GC column inhibiting detection as well as some can be lost during solvent removal [20,23]. Above all, the solvents are expensive and toxic, therefore, present safety and health issues [26]. The solvents usually employed include n-hexane, acetone, chloroform, dichloromethane, diethyl ether, aqueous methanol, pentane, mixture of pentane and diethyl ether (1:2 v/v) [25,27]. USE does not require heat and helps in the isolation of both low and high molecular weight compounds that are good potential markers for the determination of honey origin [21]. Solvent extraction methods have been used for VOC extraction, whether by means of direct extraction or SDE.

Albeit, Jerković *et al*. [25] in an attempt to develop an easier solvent extraction method, compared HD to USE for the isolation of volatile compounds from two unifloral honeys of *Robinia pseudoacacia* and *Castanea sativa* L. They revealed that USE gave the most representative profile of all honey volatiles (without artifacts) as well as it enabled the extraction of low molecular weight semi volatile markers especially benzoic, vanillic and phenyl acetic acids. Castro-Várquez *et al*. [19] equally compared three extraction techniques; SDE, LLE and solid phase extraction (SPE) that were employed in the extraction of volatiles from rosemary honey. SDE extracts were rich in terpenes and esters while the other two techniques embraced the formation of artifacts due to heating.

To avoid the generation of artifacts (furan and pyran derivatives) and also the consumption of expensive and toxic organic solvents, some new methods have been put forth recently. These methods include solid phase extraction (SPE), headspace (HS) and solid phase micro-extraction (SPME) methods. The main advantage of the HS analysis is that it is carried out on an untreated sample [28] and the profile of the isolated volatiles is closely associated with sensory perception [20]. Dynamic SPE is a technique that eliminates the use of solvent entirely and removes interfering substances such as sugars and acids by merely washing with water [29], but requires extensive modification of the gas chromatographic injector or the addition of a desorption module. Consequently, SPME resolves the handicap associated with the former technique.

SPME is a method that shows lucid advantages like; it eliminates the use of (toxic) organic solvents, allows the quantification of a large number of molecules, little or no manipulation/preparation of samples, substantially shortens the time of analysis and moreover, it is simple and covers a wide range of sampling techniques including *in situ* and air sampling [26]. Moreover, it can be easily coupled to various analytical instruments, e.g., GC, GC-MS, HPLC, LC-MS and GC-O (GC-olfactometry) [23].

However, the efficiency of SPME technique is affected by the following important parameters: fiber coating, sample amount, matrix modification by water and sodium chloride addition, agitation of sample matrix, extraction temperature, extraction time and analyte desorption [30]. All these parameters could be optimized to ensure efficient extraction in order to obtain high recoveries of honey volatiles. For example, Piasenzotto *et al*. [28] showed differences in volatile profiles of chestnut honey extracted by SPME using polydimethylsiloxane (PDMS)-coated fiber, polyacrylate (PAC)-coated fiber or Carboxen-coated fiber. Overall, PAC-coated fiber gave the best results while Carboxen-coated fiber showed peak distortion attributed to band broadening due to poor focalization inside the column; this could be related to slow desorption from Carboxen because of the high polarity of this phase. Cuevas-Glory *et al*. [16] equally indicated the advantage of using fiber composed of Polydimethylsiloxane/Divinylbenzene (PDMS/DVB). Most recently, Plutowska *et al*. [6] evaluated the extraction efficiency of different types of phases of stationary fibers employed in HS-SPME analysis of VOCs in honey; divinylbenzene/carboxen/polydimethylsiloxane (DVB/CAR/PDMS) gave the best results in comparison to the previously mentioned fibers.

SPME sampling can be performed in three basic modes: direct extraction, headspace extraction (HS) and extraction with membrane protection. HS extraction has been reported as the most appropriate sampling mode since it protects the fiber from adverse effects caused by non-volatile compounds that occurs in the sample matrix (namely, sugars) and also allows modifications (for example pH) with no effect on the fiber. Nonetheless, the drawbacks of this technique include its sensitivity against organic solvents and the limited range of stationary phase that are available commercially [26]. Several authors have used HS SPME for the extraction of VOCs from honey matrix [2,31,32]. Conclusively these varied isolation methods have been employed for the extraction of VOCs from honey matrix which were analyzed by GC-MS; valuable information were obtained that enabled the determination of honey’s botanical origins.

A novel approach for the isolation of VOCs was validated in the study by Manyi-Loh *et al*. [33]; we identified volatile compounds in solvent extracts of honeys produced in South Africa. Depending on our previous work on the antimicrobial activity of Selected South African honeys; we extracted our honeys with proven antibacterial activity with chloroform and n-hexane solvents. Subsequently, the solvent extracts were fractionated and purified by silica gel column chromatographic technique with 100% *n*-hexane or chloroform followed by the solvent systems n-hexane: ethyl acetate: acetic acid (7.8:3.5:1.25) and methanol: acetic acid: water (10:7:3) respectively. The isolated volatiles were analyzed by GC-MS technique which led to the identification of two novel compounds in honey; thiophene and N-methyl-D3-aziridine which are of great importance. Thirty other compounds belonging to the known chemical families of VOC in honey were equally identified [34].

## 3. Significance of Volatile Organic Compounds in Honey

### 3.1. Determination of Floral and Geographical Origin of Honey

Honey is often marketed as mixed-floral honey with a blend of flavors, consequently, its botanical origin which greatly influences the consumer preference, remains difficult to determine [23]. The floral origin of honey is an important characteristic in the evaluation of its quality. Indeed, the estimation of honey quality by consumers depends on its organoleptic characteristics, which is strongly dependent on its botanical origin and to some extent also on its geographical origin [2]. Honey of the same floral source can vary due to seasonal climatic changes or to a different geographical origin [3]. Monofloral or unifloral honey is produced from nectar that is derived either wholly or mainly from a single species or plant and therefore is comprised of specific VOCs [10,28]. Accordingly, VOCs could be used to discriminate between monofloral honeys from different floral origins given that the high number of VOCs gives a distinct profile that could represent the fingerprint of each honey type.

Traditionally, the botanical source of honey is determined by microscopic examination of its pollen, a technique known as melissopalinology [35]. Nevertheless, melissopalinology is expensive, time-consuming and is strongly dependent on the qualifications and judgment of the analyst [2,36]. In addition, there is the need for a pollen library; industrial infiltration may also affect the accuracy and precision of the technique [2]. Moreover, there is great variability in the nectar contribution of any particular flower compared to the quantity of its pollen found in honey. In spite of the fact that specific pollen could be present in a honey, its presence could be low in some honey types (citrus, lavender and rosemary), consequently a wider range of markers necessary to ascertain the origin of honey becomes inevitable [37].

Guyot *et al*. [38] pointed out that in some cases pollen analysis could be of no use especially when the honeys are derived from sterile plants. Castro-Várquez *et al*. [4] reported that it is more difficult to determine the floral origin of honeydew honey because palynological analysis could not be conducted considering its source of production. Therefore, there is a tendency to replace pollen analysis by profiling analytical and/or physicochemical markers for honey discrimination.

Besides the other parameters that have been extensively studied for the recognition of floral origin, VOCs is one of the main factors for differentiating honeys from different botanical/floral origin as well as geographical origins [36]. Once VOCs have been extracted by any of the aforesaid techniques and subsequently analyzed by GC-MS, typically generates a complex chromatographic profile, with multiple peaks of different intensities and the presence of characteristic compounds mixed with non-characteristic ones (mainly arising from impurities, industrial processes, a blend of different products, *etc*). From this complex profile, it is necessary to identify compounds which are characteristic of a given source, as a mandatory step to assess the origin of a food product.

With GC-MS analysis, initial identification of the compounds would be achieved by comparing their mass spectra with those of the NIST Mass Spectral Data Base [28] and or WILEY library [2]. The identity of chosen volatile compounds could be additionally verified on the basis of conformity of retention times and mass spectra with standards [35]. However, there is disagreement over which compounds could serve as floral markers for a given honey type since differences exist between plant varieties, geographical origins or beekeeping practices [4]. Also, methods of extracting the volatile fractions may display a varying degree of selectivity and effectiveness depending on the compounds involved [19].

Clearly, compounds that could be employed in the discrimination between honey’s floral origins must be focused on plant-derived compounds and their metabolites, for instance terpenes and its derivatives, benzene and its derivatives and norisoprenoids [4]. These compounds are characteristic of a given floral origin; as they are found only in certain types of honeys thus are considered as “floral markers” [39]. In other circumstances, the floral origin is determined by a greater concentration of certain compounds in some types of honey than in others or by the absence of determined compounds [14,40]. Some VOCs that have been considered as floral markers for specific honey types are shown in Table 1.

On the other hand, difficulty is encountered in finding VOCs exclusively present in some honeys obtained from a specific botanical origin; this therefore justifies the use of sensory analysis to make this differentiation possible. Sensory evaluation is one of the most useful tools employed in honey characterization since it gives complete information about honey quality [3]. The combined study of chemical and sensory variables in the characterization of monofloral honeys may improve differentiations of honey types, and also provide some insights of the consumer preferences based on honey aroma [40]. In view of that, Mannas and Altug [41] employed volatile composition together with sensory profile for estimation of authenticity of thyme honey. Knowledge about the floral origin of a honey will help to prevent fraud, standardize its quality and authenticate the honey thus allowing it to be competitive in the market [3].

Furthermore, the geographical origin of honey is a relevant factor that can influence its volatile composition. The accumulation of phytochemicals (carbohydrates, phenolic compounds and VOCs) is dependent on climatic conditions (sunlight, moisture), soil characteristics and other factors; therefore, it is reasonable to believe that differences between honeys obtained from different countries are bound to be due to the different compositions of pollen or nectar, which have the greatest influence on the chemical composition [42]. For example, the climatic conditions of Spanish chestnut forests, spread over different regions (north-east, north-west and south-east), influence the chestnut honeys volatile composition since they possess characteristic aroma profiles intimately related with the geographical area of origin [3].

### 3.2. Determination of Aroma/Flavor Profile of Honey

Flavor/fragrance qualities of honey are very much dependent on the volatile and semi-volatile organic compounds present in both the sample matrix and headspace aroma [13,30]. The aroma profile is one of the most typical features of a food product for the evaluation of both organoleptic quality and authenticity. Unifloral honey has highly characteristic aromas, indicating the presence of VOCs derived from nectar [10,38]. Volatile substances are the main factors responsible for aroma, which in concert with other factors such as taste and physical factors contribute to the flavor [2].

In general, light-colored honey is mild in flavor and darker honey has a more pronounced flavor [19,20]. Honey aroma is very complex, involving many tens of volatile compounds and is greatly dependent upon the isolation procedure employed [21]. However, not all volatile compounds have a significant impact on honey aroma.

The contribution of a given compound depends on the extent to which the concentration exceeds its odor threshold; however, the possibilities of synergistic and/or antagonistic interactions between different components should also be taken into account. Thus, even compounds present in low concentrations may contribute strongly to the honey aroma [42]. More elaborately, VOCs after being analyzed and identified by GC-MS, the concentration of each compound can be calculated from external calibration curves of standards [2].

To assess the influence of the compounds studied on overall honey aroma, odor activity values (OAV) have to be calculated by dividing the concentration of each compound by its perception threshold [4]. Of the multitude of compounds analyzed, only those displaying OAVs greater than 1 are deemed to contribute to honey aroma [23,42]. Carboxylic acids are chemical compounds that have different aromas, ranging from spicy to rancid depending on the length of carbon chain. Short chain acids such as acetic acid, have spicy flavors and aromas, while butanoic acid and hexanoic acids in butter are linked to a rancid aroma [15]. Specific VOCs with different aroma descriptions are shown in Table 2.

Conversely, an elucidation of the origin of aroma compounds should lead to a better understanding of factors causing flavor differences between honeys. In addition, to characterize the aroma of honeys from different geographical areas of origin, considerations should be made based on the variability of weather conditions, proximity of the sea and beekeeping practices [3]. Castro-Várquez *et al*. [3] differentiated chestnut honeys from north-east, north-west and south-east areas of Spain based on the significant differences in concentration of several volatile compounds and sensory characteristics pertaining to aroma.

Concisely, many consumers seek high quality products with a clear regional identity and sensory qualities associated with the areas of origin. Therefore, it is in the best interest of the apiculture industry to offer honeys with specific geographical characteristics and superior quality (aroma/flavor) to consumers. In addition, honeys from specified botanical sources often command premium price due to their organoleptic or pharmacoactive properties [45].

### 3.3. Biomedical Activities of Honey and Contribution from Its Volatile Constituents

Honey has been used in many cultures since ancient times as an effective remedy due to its therapeutic properties including anti-oxidant, anti-inflammatory, antimicrobial and immune modulatory effects [5]. Its antimicrobial activity has been attributed to four salient factors namely, hydrogen peroxide content, high osmotic pressure (low water activity), acidity (pH) and non-peroxide factors (phytochemicals). Other factors include low redox potential, low protein content (a high carbon to nitrogen ratio), bee defensin-1, viscosity (opposes convection currents and limits dissolved oxygen) and induction of increased lymphocyte and phagocytic activity [48,49].

In addition, honey is a saturated or supersaturated solution of carbohydrates of which glucose and fructose are the most abundant (84%) [50], apparently, the osmotic effect of honey is elicited owing to the fact that the strong interaction of the sugars with water molecules leaves very little or no water to support the growth of micro-organisms (bacteria and fungi since they thrive in moist environment); consequently, they become dehydrated and finally die [51]. More to that, honey is characteristically acidic with gluconic acid as the most prevalent acid that emanates from the oxidation of glucose by glucose oxidase in the presence of water and oxygen as well as from bacteria of the genus *Gluconobacter*, occasionally isolated from ripening nectar [52]. Its pH value lies between 3.2 and 4.5 due to the presence of organic acids such as gluconic, formic, acetic, propionic, hexadecanoic acid and is low enough to inhibit the growth of most pathogenic organisms whose optimum growth pH normally falls between 7.2 and 7.4 [53].

Of all the factors, the major contributor to antimicrobial activity is hydrogen peroxide; it is produced from the oxidation of glucose by glucose oxidase upon dilution of honey [54]. The slow release of this compound is mild and does not damage tissues but has antimicrobial activity due to the formation of free radicals such as hydroxyl and superoxide. Its concentration is determined by the relative levels of glucose oxidase synthesized by the bee and catalase originating from flower pollen [55]. More elaborately, major variation observed in the overall antibacterial activity between honey types is due to variation in the level of hydrogen peroxide that arises in honey and, in some cases, to the level of non-peroxide factors. However, hydrogen peroxide can be destroyed by heat or light or by components of honey; it can be degraded by reaction with ascorbic acid and metal ions and the action of enzyme catalase which comes from the pollen and nectar of certain plants [54]. In such circumstances, the antimicrobial activity of honey could be lessened or eliminated.

Despite the negative implication of catalase, light and heat on the peroxide generating system, some honeys still retain their antimicrobial activity. They are known as non-peroxide honeys, e.g., Jelly bush (*L. polygalifolium*) and Manuka (*L. scoparium*) possessing non-peroxide antibacterial factors (phytochemicals). Phytochemicals are secondary metabolites that confer color, flavor and defense against infections to plants; they are endowed with antimicrobial and anti-oxidant potential [56]. Plants biosynthesize a great number of various phytochemicals, which may possess health-promoting properties, antioxidants being the major group of bioactive constituents; they are considered as natural agents, which might reduce the risk of oxidative damage in living cells. These bioactive may be transferred from plants to nectar and further to honey [57].

In honey, they can be grouped into carbohydrates, phenolic compounds (flavonoids and non-flavonoid phenolic compounds) and volatiles [42]. Accordingly, raw honey contains copious amounts of these compounds; however, their contribution in antibacterial activity is small compared to the contribution from hydrogen peroxide [55]. Albeit, their antibacterial activity should not be overlooked since the composition of phytochemical has an influence on the biological activity of honey; usually, the same compounds have antioxidant and antimicrobial activity therefore may contribute in the efficacy of honey in therapeutic uses. It is possible therefore to assert that the antimicrobial factors (peroxide and non-peroxide) in honey could be acting in synergy in a bid to inhibit the growth of micro-organisms.

Volatiles in honeys occur most frequently in very low concentrations and are a complicated mixture of substances with various physicochemical properties and levels of stability. On that account, it is especially essential to select a suitable extraction technique [6]. In a nutshell, the bioavailability of these compounds would depend on the extraction technique which determines the type of compounds isolated as well as influences the concentration yield.

For example, Jerković *et al*. [31] investigated VOCs in *Amorpha fruticosa* honey samples using two methods, HS-SPME and USE. The HS of the honey was dominated by 2-phenylethanol (38.3–58.4%), whilst 2-Phenylethanol (10.5–16.8%) and methyl syringate (5.8–8.2%) were the major compounds of ultrasonic solvent extracts. Furthermore, the scavenging abilities of the series of concentrations of honey ultrasound solvent extracts and the corresponding honey samples was tested by a DPPH (1,1-diphenyl-2-picrylhydrazyl) assay. Approximately 25 times lower concentration ranges (up to 2 g/L) of the extracts exhibited significantly higher free radical scavenging potential with respect to the honey samples.

In another study investigating the VOCs of two oak honeydew honeys, Jerković *et al*. [58] reported terpenes as the most volatile organic headspace compounds. Also, the antioxidant capacity (FRAP assay) of honeydew samples was 4.8 and 16.1 mmol Fe^2+^/kg, while the solvent mixture extracts showed antioxidant activity of 374.5 and 955.9 Fe^2+^/kg, respectively. In addition, the ascorbic acid content of honey ranges from 0.5 to 6.5 mg/100 g with an average of 2.4 mg/100 g or 5 mg/mL. However, some specific varieties of honey have been reported to contain as much as 75–150 mg ascorbic acid per 100 g, while most honey contain less than 5 mg/100 g. The gastrointestinal absorption of ascorbic acid occurs through an active transport process, as well as through passive diffusion [59].

Maillard reaction, a non-enzymic browning reaction occurs between sugars and free amino acids during thermal treatment of honey. It is believed that Maillard reaction products (MRPs) are acting as antioxidants [60]. Hydroxymethylfurfural (HMF) is one of the major intermediate products of Maillard reaction which is formed from the condensation of sugars in the presence of an amino acid during processing, prolonged storage or excessive heating of honey [61]. Its maximum concentration is 15 mg/kg in honey and is regulated by law. Both compounds (ascorbic acid and HMF) are known antioxidants in honey with the potential to scavenge for free superoxide and reactive oxygen metabolites (radicals).

In a study conducted by Turkmen *et al*. [60] on the effects of prolonged heating on antioxidant activity and color of honey reported that the antioxidative activity increases linearly with increasing temperature. This observation was previously demonstrated by the work of Anthony *et al*. [62] on the antioxidative effect of MRPs formed from honey at different reaction times. Furthermore, Helyes and Lugasi [63] in a study to explore the antioxidative characteristic of tomato fruit showed a significant linear correlation of antioxidative potential with lycopene, polyphenol and hydroxymethylfurfural content that increased continuously during ripening period. Other MRPs namely maltol (3-hydroxy-2-methyl-4Hpyran-4-one), isomaltol [1-(3-hydroxy-2-furanyl)ethanone], 5-hydroxy, 5,6-dihydromaltol, 4-hydroxy-5-methyl-3-(2*H*)-furanone are potential precursors for antioxidants [62].

Overall, the components in honey responsible for its antioxidative effect are flavonoids, phenolic acids, ascorbic acid, catalase, peroxidase, carotenoids, amino acids, MRPs and others. It is worth mentioning that free radicals cause oxidative damage to lipid, protein, and nucleic acids which may lead to many biological complications including carcinogenesis, mutagenesis, aging and atherosclerosis. This anti-oxidant activity may be at least part of what is responsible for its anti-inflammatory action because oxygen free radicals are involved in various aspects of inflammation [64]. Taking into consideration the source-specific honey volatiles three principal categories are known *viz*. terpenes and its derivatives, norisoprenoids, and benzene derivatives [4].

#### 3.3.1. Terpenes

Terpenes are a small and heterogeneous class of naturally occurring compounds that serve as important synthetic building blocks for the production of flavors, fragrance, pharmaceuticals and nutraceuticals. They are oligomers or polymers of isoprene and can be categorized depending on the number of isoprene units into monoterpenes, sesquiterpenes, diterpenes, triterpenes and tetraterpenes. With respect to occurrence in nature, the monoterpenes represent the most important and best investigated class of terpenic compounds.

Biochemical modifications such as oxidation or rearrangement of monoterpenes produce the related monoterpenoids [65]. Quite a few honey samples have been investigated and the following monoterpenes and monoterpenoids were identified: linalool and its derivative, β-terpineol, dihydrocitronellol, β-citronellol, citronellal, geranyl acetone, limonene, β-pinene, tetrahydrogeraniol, cavacrol, p-cymene, 1,8-cineol, camphor, isoborneol, p-cymenol *etc*. [29,30]. They are known to be active against a wide range of micro-organisms including Gram negative and positive bacteria, viruses as well as fungi [66,67].

This class of compounds is also known for its biological activities in humans; limonene prevents mammary, liver, lung and other cancers acting in the promotion/progression stage [68]. Besides, their antimicrobial activity, these compounds possess antiseptic and disinfectant qualities and also very great stimulating therapeutic properties; however, they could cause side effects such as skin and mucous membrane irritation and toxicity (www.betterlivingessentials.com/pure-essential-oils/essential-oils).

#### 3.3.2. Norisoprenoids

They are substances that could be derived from the direct degradation of carotenoid molecules. Examples are α-isophorone, β-isophorone, β-damascenone and 4-oxoisophorone [69]. These molecules have an important sensorial impact on honey aroma as they have very low olfactory perception thresholds [70]. The presence of these compounds is positive indicators for their precursors, carotenoids which have been linked to the oxidation-preventing mechanisms. They are efficient free-radical scavengers and they enhance the vertebrate immune system as they possess potential antioxidant capacity [71].

#### 3.3.3. Benzene Derivatives

Volatile benzene derivatives are important since many are dominant in some honeys. Examples of such compounds include benzene acetaldehyde, benzaldehyde, 1-phenylbutane-2, 3-diol, 1, 4-dihydroxybenzene, benzene ethanol *etc*. [72,73]. Some of these compounds demonstrate antibacterial activity; methyl 4hydroxy-3, 5-dimethoxy benzoate and methyl 3, 4, 5-trimethoxy benzoate were the two major antibacterial compound detected in New Zealand native honeys [74].

Most interestingly, we identified thiophene and *N*-methyl-D3-aziridine compounds among the volatile compounds in solvent extracts of selected South African honeys [34]. These small molecules are heterocyclic compounds that serve as essential precursors in the synthesis of natural products and pharmaceuticals endowed with vital biomedical activities including, antiviral, antibacterial, antifungal, antitumor and anti-immuno-modulatory effects [75]. In addition, VOCs in honey could be used as a marker of environmental pollution. Bentivenga *et al*. [76] reported the presence of aromatic hydrocarbons which represents an indication of environmental problem, in honey obtained from Basilicata thus were used to monitor environmental pollution caused by oil extraction.

## 4. Conclusions

Honey is a very complex product, because its properties and composition depend not only on the nectar-providing plant species, but also on other factors such as bee species, geographic area, season, mode of storage, and even harvest technology and conditions [42]. However, honeys produced from different floral sources may have distinctly different aromas and tastes due to differences in volatile composition which in turn is dependent on the extraction methods and also on the botanical and geographical origins [58].

VOCs greatly influence the aroma as well as botanical and geographical origin identification of honey. Consequently, honeys from defined botanical and geographical origins possess distinctive organoleptic characteristics and as such are considered as premium products which are often traded at higher prices than honeys from mixed botanical origins. If additional properties such as the antimicrobial properties are stated by the exclusivity of the honey source, the price of the product is further increased [77]. Thus orange blossom or acacia honeys are sold worldwide at higher prices than other types of honey.

Investigation of volatile compounds of honey to date have given little importance to the correlation between chemical analysis (instrumental) and sensory analysis. Accordingly, the correlation of sensory analysis with the instrumental data has gained credibility and space in recent years, as it allows classifying products according to certain criteria and allows you to predict the sensory quality from measures. In a nutshell, knowledge on the VOCs profile in honey help to standardize the quality of honey, avoid fraudulent mislabeling of inferior products, as well as aid in authenticity of the product. The antimicrobial properties of honey could be attributed to its physical characteristics and chemical constituents [5]. Specific VOCs of honey are endowed with beneficial health properties and as such could contribute in overall biomedical activities of honey. This aspect is receiving adequate and profound attention in our laboratory.

## Figures and Tables

**Table 1:** Specific floral markers used to determine the botanical origin of particular honey types.

Method Employed	Honey Type/Country	Floral Marker	Reference

USE-GC-MS	Thyme (Greece)	3-hydroxy-4-phenyl-2-butanone and 3-hydroxy-1-phenyl-2-butanone	Alissandrakis *et al*. [43]

SDE-GC-MS	Oak honeydew (Spain)	Trans-Oak lactone	Castro-Várquez *et al*. [4]

HS-SPME-GC-MS	*Tolpis virgata*	3,5-dihydroxytoluene and tridecane	Odeh *et al*. [44]
	*Thymus capitatus*	1,3-diphenyl-2-propanone, (3-methylbutyl)benzene, 3, 4, 5-trimethoxybenzaldehyde and 3, 4-dimethoxybenzaldehyde	
	*Thymalae hirsuta*	Benzene propanol, benzylalcohol, nonanal, hexanol and 4-methoxyphenol	
	(Palestine)		

HS-SPME-GC-MS-Olfactometry	Acacia	Hexanal	Wardencki *et al*. [32]
	Buckwheat	Pentanal, furfural and 2-ethylhexanol	
	Lime	*p*-methyl acetophenone	
	Honeydew	Methyl butanal and lilac ldehyde	
	Rape	*p*-Cymene	
	(Poland)		

SPME-GC-MS	Citrus	Methyl anthranilate and limonene diol	Piasenzotto *et al*. [28]
	Lime tree	*p*-methyl acetophenone, carvacrol and 8-*p*-menthen-1,2-diol	
	Thyme	Ethenyl phenyl acetate	
	Dandelion	Phenyl acetonitrile	
	Chestnut	Aminoacetophenone	
	Eucalyptus	Nonanoic acid and acetoin	
	(Italy)		

USE-GC-MS	*Salvia officinalis* L. (Croatia)	Benzoic acid and phenyl acetic acid	Jerković *et al*. [13]

HS-SPME-GC-MS	*Medicago sativa* (alfafa)	Octanal, benzene acetaldehyde,1-octanol, 2-methoxyphenol, nonanal and 2-H-1-benzopyran-2-one	Baroni *et al*. [2]
	*Helianthus annus* (sunflower)		
	*Melilotus albus* (white clover)		
	*Prosopis* spp (carob)		
	*Prosopis caldenia* (Calden)		
	(Argentina)		

LNSDE-SE-GC-MS	*Calluna vulgaris* (France, Belgium, Uk, Norway, Germany)	Phenyl acetic acid, dehydrovomifoliol and 3,5,5-trimethylcyclohex-2-ene derivative	Guyot *et al*. [38]
	*Erica arborea* (France, Greece, Italy)	4-methoxybenzaldehyde, 4-methoxybenzoic acid And methyl vanillate	

SPME-GC-MS	*Quillajia saponaria* (Quillay)	Megastigmatrienone, 2-*p*-hydroxyphenyl alcohol, β-pineme and linalool oxide.	Montenegro *et al*. [11]
	*Escallonia pulverulenta* (corontillo)	Safranal	
	*Eucryphia cordifolia* (Ulmo)	Isophorone and cetosiophorone	
	(Chile)		

MSDE-GC-MS	Eucalyptus	3-caren-2-ol, p-cymene and its derivate alcohol.	Castro-Várquez *et al*. [40]
	Citrus	Linalool oxide, lilac alcohol and lilac aldehyde	
	Lavender	Nerolidol oxide	
	(Spain)		

**Table 2:** Aroma description of some volatile organic compounds in honey.

Volatile Compound	Aroma Description	Reference

Nonanal	Aldehyde, citrus, fatty floral Green, spiny	Bayraktar and Onoğur [46]
Nonanol	Green, sweet, oily	
Decanal	Soap, orange, peel, tallow	
Octanal	Fat, soap, lemon, green	

Linalool	Sweet, citrus, forest, geranium	Wardencki *et al*. [32]
Benzaldehyde	Sweet, almond, marzipan	
Dimethyl sulphide	Sweet, honey, acrid, cooked vegetables, sulphuric	
Furfural	Sweet, fruit, cherry soft almond	

Sinensal (isomer I)	Sweet, orange	Castro-Várquez *et al*. [29]
Sinensal (isomer II)	Sweet, orange	
β-damascenone	Fruity, sweet, honey	
Phenylacetaldehyde	Sweet, honey-like	

Isophorone and cetoisophorone	Spicy	Montenegro *et al*. [11]

Benzene and phenolic acids	Ripe fruit and spicy	Castro-Várquez *et al*. [40]
Hexanol and hotrienol	Balsamic and aromatic herb	
3-caren-2-ol and spathulenol	Cheese and hay	

γ-butyrolactone, pantolactone, and oak lactone	Woody, toasty, caramel	Cullere *et al*. [47]
